# Outcomes of Kidney Transplantation by Using the Technique of Renal Artery Anastomosis First

**DOI:** 10.7759/cureus.3223

**Published:** 2018-08-28

**Authors:** Zi Qin Ng, Wai Lim, Bulang He

**Affiliations:** 1 WA Liver & Kidney Transplant Service, Sir Charles Gairdner Hospital, Perth, AUS; 2 Nephrology, Sir Charles Gairdner Hospital, Perth, AUS

**Keywords:** transplant surgery, arterial anastomosis, kidney transplant, complication

## Abstract

Introduction

The surgical technique for kidney transplantation has been well established: the renal vein is anastomosed first, followed by renal artery anastomosis. Alternatively, the renal artery can be anastomosed first and then the renal vein for kidney transplantation. However, there is a lack of data on the outcomes of kidney transplantation by using this alternative approach. The objective of this paper was to review the outcomes of kidney transplant by using this approach.

Methods

A review of 205 consecutive kidney transplants was conducted. All kidney transplants were performed by doing renal artery anastomosis first and then the renal vein. Data were collected, including vascular/urological complications and kidney graft function.

Results

All transplants were performed successfully with no occurrence of renal artery/vein thrombosis and urine leakage. There were five cases of renal artery stenosis that were managed with endovascular intervention. There was no recurrence on follow-up. One ureteric stenosis required surgical reconstruction.

Conclusions

This alternative vascular anastomotic technique is efficient and safe. It avoids flip-flopping the kidney graft during the vessel anastomoses and may be more practical in minimally invasive surgery for a kidney transplant due to the space constraint.

## Introduction

Kidney transplantation is the preferred treatment for patients with end-stage kidney disease because it confers a survival advantage and improves quality of life [1]. The incidence of surgical complications after kidney transplant has improved over the last two decades, from as high as 30% to less than 6% currently, which is attributed to the refinement of operative technique in kidney procurement, preparation, and transplantation [2]. One of the most feared complications early post-transplant is vascular thrombosis of the renal artery or vein [2], which is associated with an up to 5% greater relative risk of graft loss [3]. Predisposing factors for renal vascular thrombosis are multifactorial but usually related to a combination of donor, recipient, transplant, and/or surgical-related factors.

The basic technique of a kidney transplant surgery involves vascular and ureteric anastomoses. Vascular anastomosis of the renal artery to the external or internal iliac artery and the renal vein to the external iliac vein is a critical part of transplant surgery, which should be completed within an hour to minimize the potential deleterious effect of prolonged warm and cold ischaemic time on kidney allograft function [4]. The common approach is to anastomose the renal vein first, followed by anastomosis of the renal artery [5]. This technique has been adopted universally since the 1950s. The complication rates of early renal vessel thrombosis and late renal vessel stenosis of this conventional approach were reported up to 6% and 23% [2,6-10]. An alternative surgical approach is to anastomose the renal artery first followed by the renal vein, the so-called “artery first, vein second” approach (AFVS). The efficacy and the incidence of surgical complication following this alternative surgical approach has not been reported in the literature. Therefore, the aim of this study was to review the outcomes of kidney transplant by using the technique of AFVS in a single center.

## Materials and methods

Study population

The retrospective review was approved by the Quality improvement committee (NSQHSS/EQuIP 14296) of the institute and conformed to the ethical guidelines of the 1975 Helsinki Declaration. A total of 205 consecutive kidney transplant recipients between January 26, 2010, and May 30, 2014, were included in this review.

Data collection

A retrospective review of the medical records and electronic data of all kidney transplant recipients was undertaken. Data collected encapsulated donor factors, including donor type, i.e., deceased donor: brain-dead donor, circulation-ceased donor, and live donor; and urologist referral of tumor-excised kidney grafts for transplant, site of donor kidney, recipient factors, including demographic details, number of previous transplants, and site of kidney transplant; and transplant-related factors, including ischaemic time and surgical technique. The outcomes of interest included early vascular complication that was defined as a renal artery or vein thrombosis within seven days post-transplant, a late vascular complication that was defined as a renal artery or vein stenosis during follow-up, and allograft function as measured by serum creatinine (mmol/L) at one month and last available.

Surgical technique

**Figure 1:**
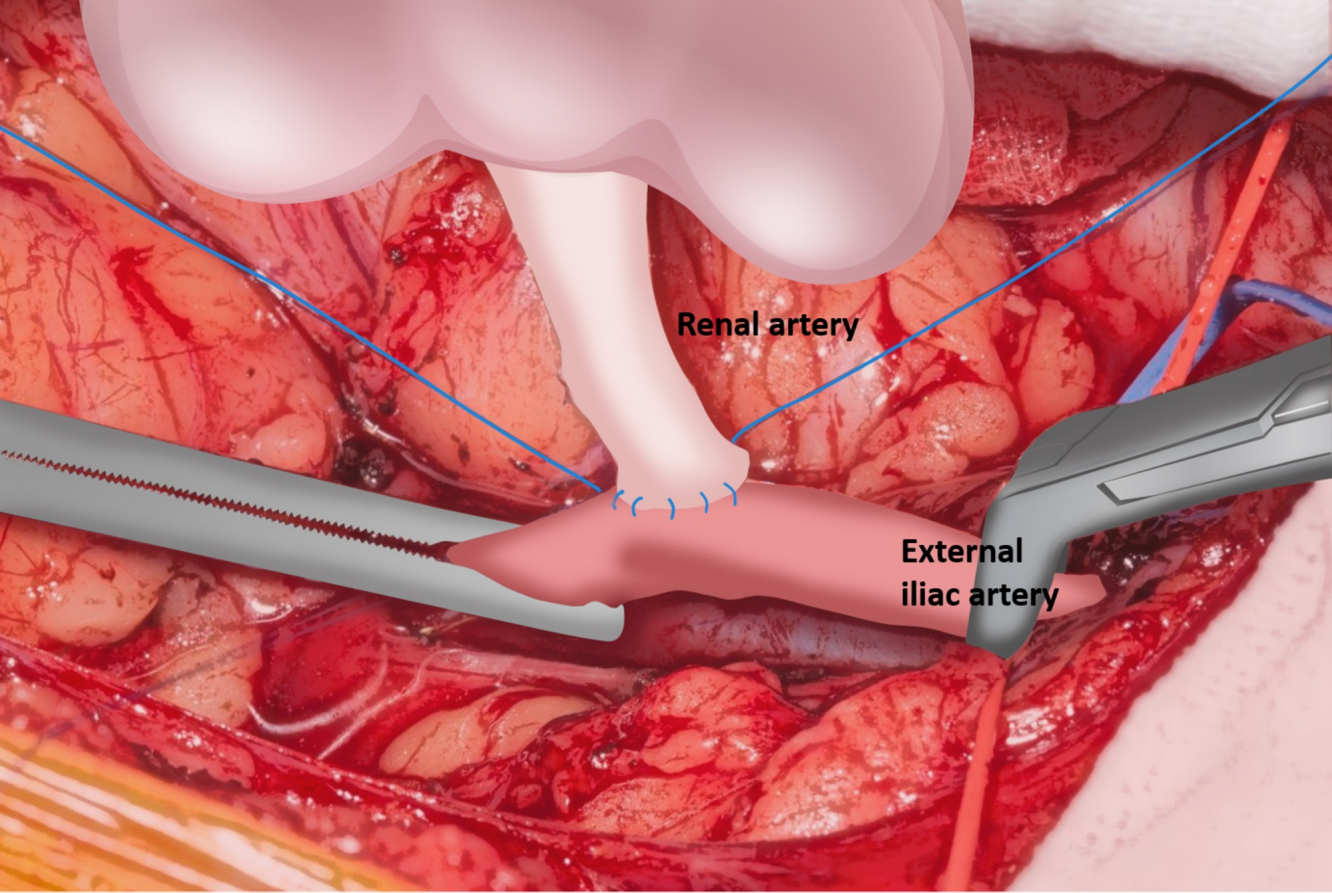
Renal artery anastomosis to the external iliac artery is commenced.

A deceased donor kidney was retrieved by using the national standard technique for multi-organ retrieval. A live donor kidney was procured by laparoscopic surgery. All except one kidney transplant were performed by the open surgical approach [11]. In brief, the kidney allograft was routinely prepared on the back table prior to transplant surgery. In general, for the left kidney from a deceased donor, the aortic patch is preserved for the renal artery to external iliac artery anastomosis. When the right kidney was used from a deceased donor, either the renal vein was extended by using the vena cava or the renal artery was shortened to ensure that the renal artery and renal vein were comparable in length. Following the preparation of the patient, a Rutherford-Morison incision was made at the right or left iliac fossa to access to the iliac vessels. Heparin (2500-4000 IU bolus) was given intravenously prior to the clamping of the iliac vessels. The renal artery was first anastomosed in an end-to-side fashion to the external iliac artery. The corner sutures (6/0 Prolene) were placed while the kidney allograft was first placed at the medial side of the incision. The lateral side of the renal artery was anastomosed by continuous suture using the 6/0 Prolene suture. The kidney allograft was then flipped to the lateral side and the medial side of the renal artery was anastomosed to the external iliac artery. The anastomosis was checked by placing a small vascular bulldog to the renal artery and the vascular clamp was released over the external iliac artery (Figure 1).

**Figure 2:**
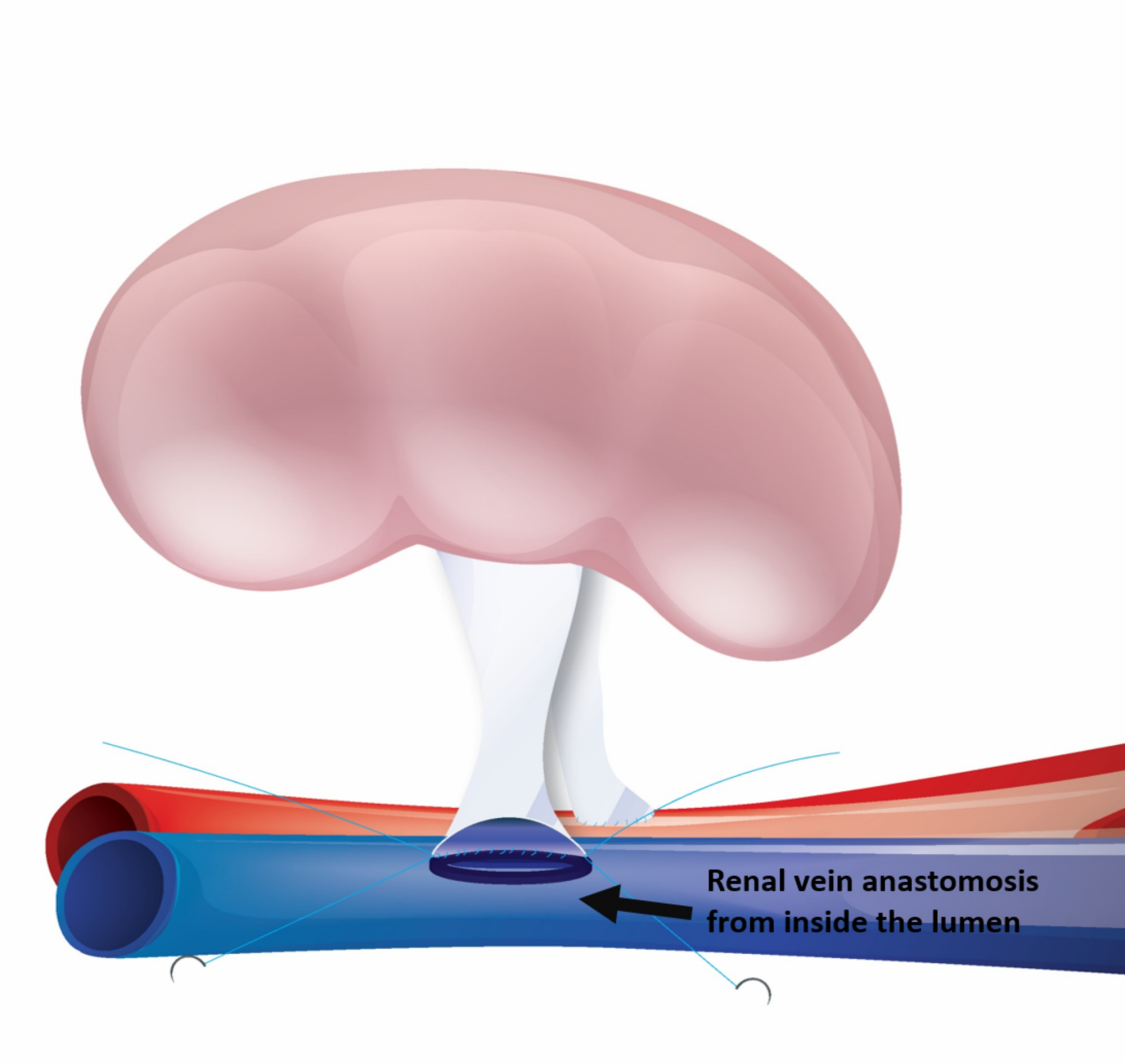
After completion of renal artery anastomosis, the renal vein anastomosis commenced from inside the lumen while retaining the kidney in the same position.

Following the renal artery anastomosis, the renal vein was anastomosed in an end-to-side fashion to the external iliac vein. Two corner sutures (5/0 Prolene) were placed first, then the lateral side of renal vein anastomosis was performed with continuous sutures from inside the lumen, and then a medial side anastomosis was performed by continuous sutures from the outside of the lumen (Figure 2). Similarly, the anastomosis was checked by placing a vascular clamp over the renal vein and then releasing the vascular clamp on the external iliac vein.

**Figure 3:**
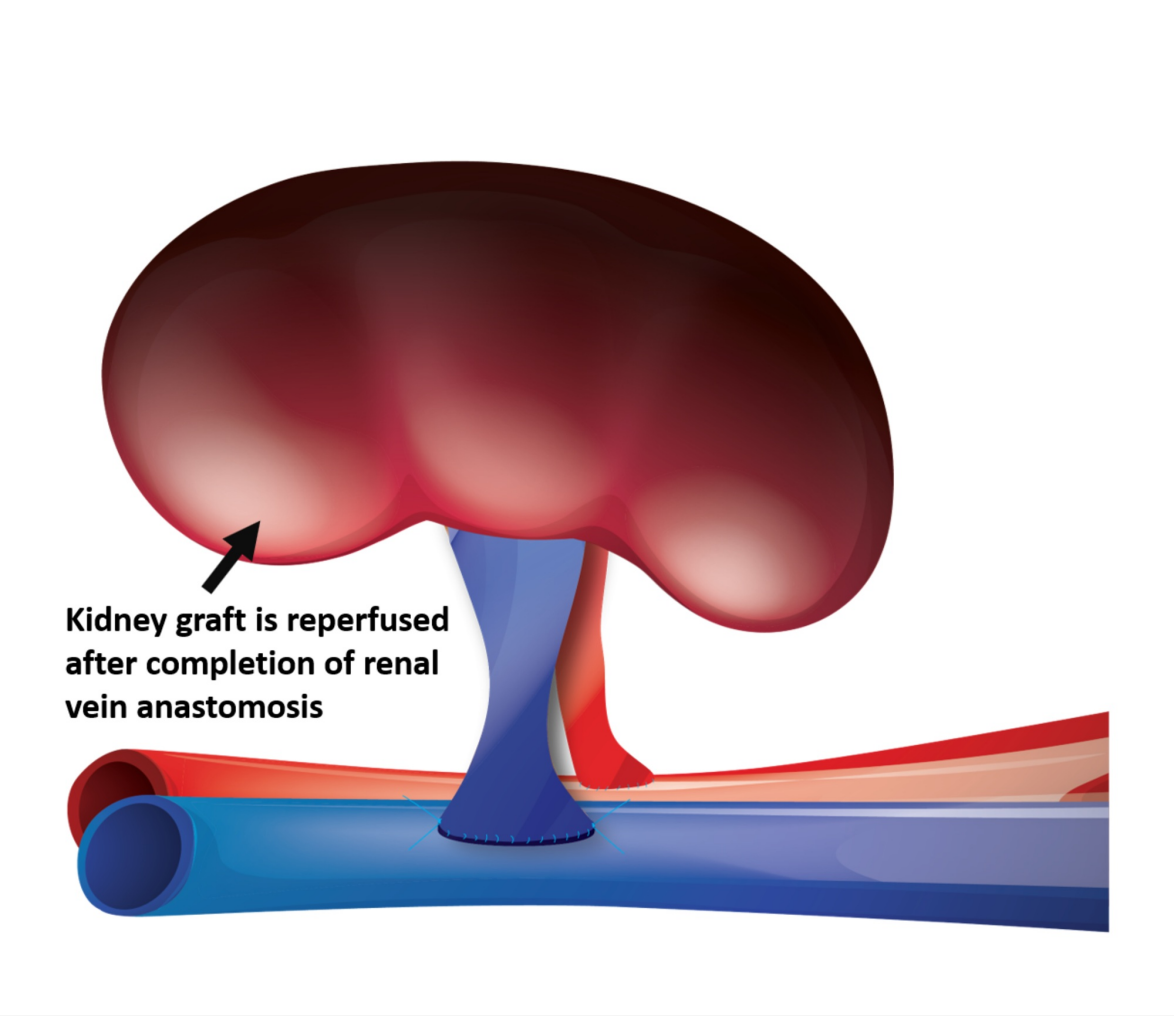
The renal vein anastomosis has been completed. The kidney graft is reperfused.

Kidney allografts with multiple renal arteries were either reconstructed on the back table or separate anastomoses were performed. The kidney allograft was then reperfused by releasing the venous vascular clamp first followed by releasing the renal artery clamp (Figure 3).

Ureteroneocystostomy was performed by using the Lich-Gregoir technique with a 5/0 polydioxanone suture. A ureteric stent (6 Fr X 12 cm) was routinely placed in-situ. A 15 Fr Blakes drain was placed and the wound was closed in layers.

Dual kidney transplantation technique

The detailed technique for a dual kidney transplant has been described previously [12]. Dual deceased donor adult kidneys were retrieved using the usual method and prepared separately on the back table, whereas dual kidneys from pediatric deceased donors were retrieved en-bloc along with the distal donor aorta and inferior vena cava. The kidneys were transplanted unilaterally with one graft placed superiorly and the other inferiorly or en-bloc using a standard Rutherford-Morison incision. AFVS was used for the vascular anastomosis and the ureter was spatulated with one edge sutured together, forming one spatula for ureteroneocystostomy by the Lich-Gregoir technique.

Tumor-resected kidney transplantation technique

The use of a small tumor-resected kidney allograft from patients with a small (less than 3 cm) renal cell tumor for a kidney transplantation has been implemented in our unit since 2007 [13]. The tumor was excised and frozen section was performed to ensure that the margin was clear prior to transplant. The excised area was repaired by oversewing the small vessel stumps and the opening of the collecting system. The kidney allograft was then transplanted with the AFVS approach and the Lich-Gregoir technique for ureteroneocystostomy.

Post-transplant care

The immunosuppressive regimen was a triple therapy with or without basiliximab induction (20 mg Day 0 and Day +4). The recipient was given a subcutaneous injection of heparin 5000 IU twice a day for thromboembolism prophylaxis. A baseline Doppler ultrasound and nuclear perfusion scan (technetium-99m mercaptoacetyltriglycine (99mTc MAG3)) of the kidney allograft was performed on Day 1 post-transplant for all patients as our routine practice. A Doppler ultrasound was repeated when clinically indicated. The ureteric stent was removed by flexible cystoscopy between four and six weeks after transplant. Computed tomography angiogram of the kidney allograft or digital subtraction angiogram may be performed if there is any clinical suspicion of transplant renal artery stenosis. Cystoscopy and retrograde pyelogram or percutaneous nephrostomy and pyelogram were performed if there were clinical suspicion of obstructive uropathy.

Statistical analysis

The baseline characteristics of the cohort were expressed as number (proportion) or mean (±SD) for categorical and normally distributed, respectively. The data were analyzed on an Excel spreadsheet (Microsoft Office 2016, Microsoft Corporation, Washington, US) and is expressed as mean (±SD) or median (IQR).

## Results

All 205 kidney transplants were successfully performed by using the AFVS approach. Of the 205, 136 (66%) recipients were male and the mean ± SD age was 50±15 years. Table 1 shows the baseline characteristics of the study cohort. The majority of the kidney transplants were deceased donor allografts (n=123) (60%), 69 (33.5%) were live-donor allografts, and 13 (6.5%) were tumor-resected kidney allografts. There were six (2.9%) cases of dual-kidney transplants. One hundred and fifty-one kidney allografts were transplanted in the right iliac fossa while 55 allografts were transplanted in the left iliac fossa. Data for the site of donor kidney allografts were available in 134 (65.4%) recipients. Twenty-five and 10 right donor kidney allografts were placed in the right iliac fossa and left iliac fossa, respectively, whereas 82 and 17 left donor kidney allografts were placed in the right iliac fossa and left iliac fossa, respectively. The mean ± SD vessel anastomotic time was 51±11.53 minutes (range: 27 to 90 minutes (dual transplants)), whereas the mean ± SD cold ischaemic time was 418±305.73 minutes (range: 40 – 1764 minutes).

**Table 1:** Patient demographics, intraoperative and postoperative details.

Characteristics	Number
Gender (Male:Female)	136:69
Mean age (Years)	50 ± 15.46 (3-81)
Deceased donor (DBD, DCD)	123
Living donor	69
Tumor-resected kidney	13
Position of transplant (Right iliac fossa: Left iliac fossa)	152:45
Surgical technique (Open: Laparoscopic)	204:1
Cold ischaemic time (mean) (minutes)	418 ± 305.73 (40 - 1764)
Vessel anastomotic time (mean) (minutes)	51 ± 11.53 (27 - 90)
Ureteroneocystostomy time (mean) (minutes)	19.7 ± 10.76 (8 - 45)
Mean length of stay (days)	8 ± 7.2 (4 - 64)
Mean follow-up (months)	55 (30 - 82)

Allograft outcomes: vascular complications

There were no early vascular complications of renal artery or vein thrombosis post-transplantation. Five (2.4%) recipients developed a late vascular complication of renal artery stenosis at the para-anastomotic site, with a median time to diagnosis of 12 weeks. All cases were initially investigated with Doppler ultrasound for deteriorating graft function. Of these five cases, four kidney allografts were from deceased donors and one was from a live donor. All five cases of renal artery stenoses were successfully managed by percutaneous transluminal angioplasty and stents without complications. The technical and clinical success rate was 100%. After a median follow-up period of 60 months, there was no recurrence of renal artery stenosis.

One allograft was lost due to dehiscence at the renal artery anastomotic site five days post-transplant, more likely attributed to the infection. No cases of hematomas that required intervention were observed.

Allograft outcomes: urological complications

One recipient from a live donor developed distal long segment ureteral stenosis after three months post-transplant, which required surgical reconstruction after a poor response to interventional radiology balloon dilatation. There were no cases of urine leakage.

Allograft outcomes: graft function

Forty-eight recipients (38%) experienced delayed graft function, defined as requiring dialysis within the first week post-transplant. All cases of delayed graft function occurred in deceased donor kidneys. The mean ± SD creatinine level was 148±183 mmol/L at one-month post-transplant. The mean ± SD creatinine level was 157±115.33 mmol/L during the median follow-up of 55 months (range 30-82 months).

## Discussion

From our cohort study, it was observed that the outcomes of kidney transplantation are satisfactory by using the AFVS vascular anastomosis technique. We have found that the renal artery first approach allows the renal artery to be anastomosed in a delicate fashion under better exposure. This approach is associated with the advantage of maintaining the kidney allograft in one position during vessel anastomoses, whereas the renal vein first approach will require a repositioning of the kidney allograft during the vascular anastomoses. Less manipulation of the kidney allograft is desirable and may reduce the error of vessel kink. Most importantly, no renal artery or renal vein thrombosis occurred nor was urine leakage encountered. Furthermore, there was a low incidence of renal artery stenosis (2.4%) and ureteric stenosis (0.49%) during follow-up in this cohort. It was also learned that the AFVS approach is feasible for all donor types, including dual kidney, tumor-resected, and en-bloc kidney transplants with a low incidence of vascular complications. However, it is understood that the technique of renal vein anastomosis first is a widely adopted technique for kidney transplantation. The technique of AFVS has achieved comparable outcomes in kidney transplantation.

In this study, it has been shown that the renal artery first technique is associated with a low risk of vascular complications. Of particular note, there were no cases of renal artery or vein thrombosis, which has been reported in up to 4% [8] to 6% and often result in kidney graft loss in the conventional renal vein first approach [9-10]. It is postulated that the conventional renal vein first approach could potentially cause excess manipulation and flip/flopping of the kidney graft during vessel anastomoses, predisposing to renal artery and/or vein kinking and thus thrombosis.

Transplant renal artery stenosis is a relatively frequent late vascular complication following a kidney transplant. The major cause of transplant renal artery stenosis is due to the intima injury that may occur during organ retrieval or at transplantation and is often located at the para-anastomotic site [14]. Multiple factors have been shown to contribute to transplant renal artery stenosis, including mechanical (e.g. kinking of the artery, suturing technique error), immunological factor (e.g. chronic rejection) [15] and infection (e.g. cytomegalovirus) [16] causes. The incidence of transplant renal artery stenosis in this cohort was 2.4%, which is on the low side of literature reports, ranging from 1%-23% [2,17-18]. The treatment for transplant renal artery stenosis is in favor of endovascular intervention. The surgical reconstruction is only required in very rare circumstances [2]. In our cohort, endovascular intervention led to successful resolution of the stenosis and improvement of allograft function.

The episode of delayed graft function is not an uncommon event after a kidney transplant, ranging from 19% to 25%, with a higher incidence in deceased circulation death kidney transplants [19-20]. A few studies have demonstrated that a prolonged ischaemic time predisposes the risk of delayed graft function. A study using the ANZDATA registry showed that recipients with more than 14 hours of total ischemia time were more likely to experience a delayed graft function and this was also associated with a 9% increase in overall risk of graft loss per hour increase in total ischaemic time [19]. Similarly, a French study showed every additional hour of cold ischemia time was associated with a 1.013 times increased risk of graft failure and a 1.018 times increased risk of death [21]. The artery first technique avoids the maneuver of the flip/flop of the kidney graft during the vessel anastomoses, as such to reduce the injury of the rewarming effect and the anastomotic time is within acceptable limits and comparable to the literature of less than 60 minutes in conventional vessel anastomoses [22].

From our observation, it has been shown that this alternative vascular anastomotic technique is safe, with a satisfactory outcome of kidney transplant compared to the conventional technique for a kidney transplant. Recently, there is a blossoming interest in developing minimally invasive techniques for kidney transplantation [23], including robotic-assisted kidney transplantation [24] and laparoscopic-kidney transplantation [11,25]. In these minimally invasive techniques for kidney transplantation, the main challenge is to perform a quality vascular anastomosis in a restricted workspace. This alternative AFVS vascular anastomotic technique could be applied in this setting to help overcome the technical difficulty.

The limitation of this study is a retrospective without a control group. The details of renal artery numbers and conditions were not recorded accurately when reviewing the record. The anastomotic time for arteries and veins was not calculated separately. A further study with a randomized control trial would be beneficial to demonstrate which technique is superior to another. However, randomized controlled trials comparing an alternative approach for vascular anastomosis is unlikely to be feasible and, therefore, the assessment of alternative surgical techniques will be confined to retrospective studies. Our review is the first that has evaluated the outcome of the AFVS approach for kidney transplantation in all types of kidney transplants from different categories of donors.

## Conclusions

In summary, this single-center experience has shown that an alternative “artery first, vein second” anastomotic technique for kidney transplants is associated with low rates of early post-transplant vascular and urological complications. The kidney graft function is satisfactory and comparable to those with conventional vein first vascular anastomosis. This is also the first cohort study to provide the evidence that the renal artery first anastomosis has a comparable outcome in comparison with the customary renal vein first anastomosis in kidney transplantation. The utilization of this technique could be considered in minimally invasive techniques for kidney transplantation, such as robotic-assisted kidney transplantation and laparoscopic-kidney transplantation, where minimal manipulation of the kidney allograft is often desirable.
